# Unnecessary thyroid surgery rate for suspicious nodule in the absence of molecular testing

**DOI:** 10.1530/ETJ-23-0114

**Published:** 2023-10-11

**Authors:** Maria Mavromati, Essia Saiji, Marco Stefano Demarchi, Vincent Lenoir, Amanda Seipel, Paulina Kuczma, François R Jornayvaz, Minerva Becker, Eugenio Fernandez, Claudio De Vito, Frédéric Triponez, Sophie Leboulleux

**Affiliations:** 1 Department of Endocrinology, Diabetology, Nutrition and Therapeutic Education, Geneva University Hospitals, Rue Gabrielle Perret Gentil, Geneva University, Geneva, Switzerland; 2 Department of Pathology, Geneva University Hospitals, Rue Gabrielle Perret Gentil, Geneva, Switzerland; 3 Department of Endocrine Surgery, Geneva University Hospitals, Rue Gabrielle Perret Gentil, Geneva, Switzerland; 4 Department of Radiology, Geneva University Hospitals, Rue Gabrielle Perret Gentil, Geneva, Switzerland; 5 Department of Oncology, Geneva University Hospitals, Rue Gabrielle Perret Gentil, Geneva, Switzerland

**Keywords:** Bethesda III, Bethesda IV, unnecessary thyroid surgery, rate of malignancy

## Abstract

**Background:**

Molecular tests for suspicious thyroid nodules decrease rates of unnecessary surgeries but are not widely used due to reimbursement issues. The aim of this study was to assess the rate of unnecessary surgery performed in real-life setting for Bethesda III, IV and V nodules in the absence of molecular testing.

**Method:**

This is a single-center retrospective study of consecutive patients undergoing fine needle aspiration cytology (FNAC) with rapid on-site evaluation between January 2017 and December 2021. Unnecessary surgery was defined as surgery performed because of Bethesda III, IV, or V results in the absence of local compressive symptoms with final benign pathology and as second surgery for completion thyroidectomy.

**Results:**

In the 862 patients (640 females, mean age: 54.2 years), 1010 nodules (median size: 24.4 mm) underwent 1189 FNAC. Nodules were EU-TIRADS 2, 3, 4, and 5 in 3%, 34%, 42%, and 22% of cases, respectively. FNAC was Bethesda I, II, III, IV, V, and VI in 8%, 48%, 17%, 17%, 3%, and 6%, respectively. Surgery was performed in 36% of Bethesda III nodules (benign on pathology: 81%), in 74% of Bethesda IV nodules (benign on pathology: 76%) and in 97% of Bethesda V nodules (benign on pathology: 21%). Surgery was considered unnecessary in 56%, 68%, and 21% of patients with Bethesda III, IV, and V nodules, respectively.

**Conclusion:**

In this real data cohort surgery was unnecessary in more than half of patients with Bethesda III and IV nodules and in 21% of patients with Bethesda V nodules.

## Introduction

Thyroid nodules are common, being palpable in 5% of adults and present in more than 60% of adults on high-frequency ultrasound (US) of the neck (1, 2). Five to 10% of these nodules are malignant, and patients are then treated with surgery, active surveillance, or local treatment, depending on the size of the nodule (3, 4, 5, 6). Given the high rate of thyroid nodules, most of which are benign, to reduce fine needle aspiration cytology (FNAC) and unnecessary surgery, ultrasound scores are used to determine which nodules should undergo FNAC and the Bethesda classification is used to assess management based on the risk of malignancy (7, 8). Suspicious nodules with Bethesda III, Bethesda IV, and Bethesda V cytology, which represent 25–40% of all thyroid nodules and carry a risk of malignancy of 10–30%, 25–40%, and 50–75%, respectively, are often considered for diagnostic surgery. Furthermore, because malignancy is not diagnosed preoperatively, a lobectomy is the surgical procedure performed in most cases and depending on the final pathology and risk of recurrence classification, a second surgery may be necessary to complete total thyroidectomy. Preoperative molecular analyses are now available for suspicious nodules (9, 10, 11). With a sensitivity for cancer diagnosis of 91-95% and a specificity of 82–90%, their use reduces the rate of unnecessary surgery by 50% (12). Their benefit to the patient is obvious. However, they are not widely used in Europe due to their price and reimbursement issues. They are considered cost-effective in the U.S. through studies based on simulation cost analysis with theoretical models including thyroid nodule management based on guidelines and performance of molecular testing derived from clinical studies (13, 14, 15). Calculations of cost-effectiveness based on the assumption that, in the absence of molecular testing, all indeterminate thyroid nodules would be treated with diagnostic surgery are inaccurate because this assumption is erroneous. Furthermore, in this setting, if every positive molecular test leads to surgery, overtreatment could be an issue. Indeed, in a retrospective study comparing the management and cost of care of consecutive patients seen before and after the introduction of ThyroSeq v2, the rate and overtreatment only slightly decreased from 19% in the absence of molecular testing to 17% with the use of molecular testing with an overall rate of malignancy that remained equal and an average cost per thyroid cancer that increased by 47% (16). Although results would most probably differ with the use of more recent and accurate molecular tests, cost analysis should also consider follow-up after first management decisions. In order to evaluate the impact of molecular testing, assessing management in a real-world setting would be helpful.

The objectives of this study were to characterize consecutive nodules undergoing FNAC and to evaluate, in a real-life setting, the rate of unnecessary surgery performed for Bethesda III, IV, and V nodules in the absence of molecular testing.

## Materials and methods

### Study design and participants

This is a single-center retrospective study including data from consecutive patients who underwent ultrasound guided FNAC between January 2017 and December 2021 in the endocrinology and radiology division of Geneva University Hospital. Nodules diagnosed as intrathyroid metastases of nonthyroid malignancy and nodules with missing data on EU-TIRADS score and size were excluded.

The objectives of this study were to assess the rate of malignancy among nodules that underwent FNAC and among nodules that underwent surgery according to their EU-TIRADS and Bethesda results and to evaluate the rate of unnecessary surgery performed in a real-life setting for Bethesda III, IV, and V nodules in the absence of molecular testing.

The study was approved by the Swiss Ethics Committee in compliance with the Declaration of Helsinki; a waiver of informed consent was granted as the study was determined to involve no risk to the subjects included by using existing medical file information.

### FNA cytology

FNA were performed in the endocrine and radiology divisions under US guidance. Nodules were classified according to the EU-TIRADS score (8).

Rapid on-site evaluation was provided in all cases, to determine validity of samples. FNAC samples were collected to prepare four to six slides immediately fixed in methanol for conventional smears. Material remaining in the needle was rinsed and collected in CytoLyt for ThinPrep slide preparation (Hologic, INC.). All conventional and liquid-based cytological smears were stained with Papanicolaou stain. Cytological features were evaluated and reported according to the criteria defined by The Bethesda System for Reporting Thyroid Cytopathology, second edition (17).

### Surgical procedures

Indications for surgery included local symptoms and/or suspicious Bethesda III, IV, V, or VI results. In case of unilateral Bethesda III, IV, V nodules and in the absence of local compressive symptoms or voluminous contralateral nodules, a lobectomy was performed. In case of Bethesda VI result, in nodules of 2 cm or less and in the absence of abnormal neck lymph node on US, a lobectomy with a prophylactic central ipsilateral neck lymph node dissection was performed. In case of Bethesda VI result in nodules larger than 2 cm, a total thyroidectomy with central ipsilateral neck lymph node was performed. Completion total thyroidectomy was proposed after lobectomy in case of cancer larger than 2 cm or metastatic lymph nodes.

### Histology

Surgical specimens were formalin fixed and paraffin embedded. Nodules were classified according to the WHO 2017 criteria (18). For encapsulated neoplasms, the capsule was entirely submitted for histological examination. Diagnosis of noninvasive follicular thyroid neoplasms with papillary-like nuclear features (NIFTP) was based on WHO 2017 criteria (18). The regrouping of NIFTP, thyroid tumors of uncertain malignant potential (TUMP), and trabecular hyalinizing tumors within the low-risk neoplasms was done according to the WHO 2022 classification (19).

### Data review

In the case of a first Bethesda I or Bethesda III cytology with repeated FNAC, the nodule was classified according to the results of the last cytology, with the exception of a last Bethesda I result, in which case the nodule remained in its first category. If no second FNAB was performed, the nodule was classified according to the only cytology result available.

Only the pathology result of the thyroid nodule that underwent FNAB was considered: if a cancer was incidentally found on pathology in addition to the benign nodule biopsied, the specimen was considered benign.

### Evaluation criteria

The rate of malignancy for each EU-TIRADS category and each Bethesda class was calculated for nodules that underwent surgery and for all nodules that underwent FNAC. Nodules examined with FNAC without surgery were considered benign in this latter analysis. For EU-TIRADS 5 nodules, the rate of malignancy was also calculated according to the number of suspicious signs: solid hypoechoic, microcalcifications, irregular borders, taller than wide.

Unnecessary surgery was defined as surgery performed in patients without local compressive symptoms for Bethesda III, IV, or V nodules that were proved to be benign at final histology. A second surgery for completion thyroidectomy due to malignancy at initial lobectomy was also defined as unnecessary.

### Statistical analysis

A descriptive analysis was done, with baseline characteristics reported as mean ± S.D., median (interquartile range), or number (%), as appropriate. Statistical analyses were performed in SPSS version X and in Stata version 17.0 SE. The association between EU-TIRADS score and rate of malignancy in Bethesda III, IV, and V nodules was evaluated with a Fisher’s exact test. *P*-values <0.05 were considered statistically significant.

## Results

### Patients and nodules

**Figure 1:**
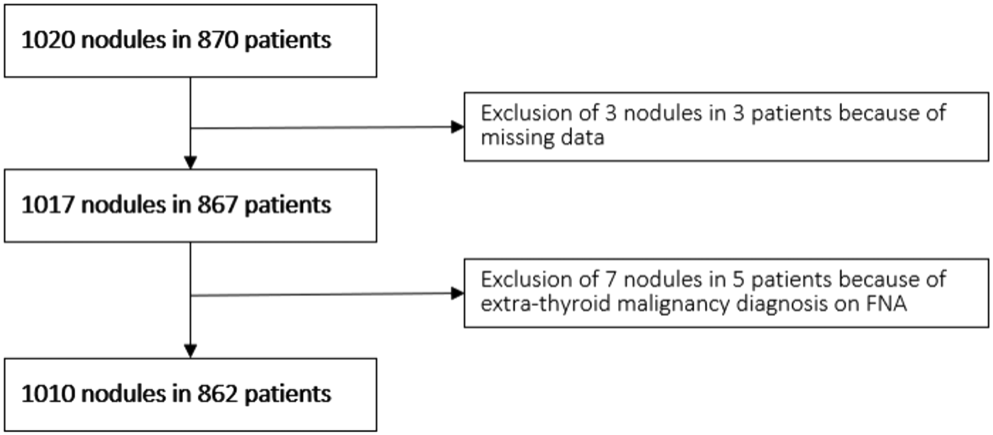
Flowchart of the nodules and patients included.

Of the 870 patients who underwent FNAC between January 2017 and December 2021, 862 met the inclusion criteria (Fig. 1). In these patients, 1189 FNACs were performed in 1010 nodules (640 females, mean age: 54.2 years; range: 12.9–92).

Nodule characteristics, including size, EU-TIRADS score, and Bethesda classification, are detailed in Table 1. EU-TIRADS scores 3 and 4 were the most common among nodules (33.5% and 41.9%, respectively), and suspicious findings on the FNAC specimen, i.e. Bethesda III (17.2%), IV (16.6%), and V (3.0%), represented 36.8% of the total specimens.

Of the 100 nodules with a first Bethesda I result, 29 underwent a second procedure classified as Bethesda I in 12 cases, Bethesda II in 7 cases, Bethesda III in 9 cases, and Bethesda IV in 1 case. Of the 228 nodules with a first Bethesda III result, 114 underwent a second procedure classified as Bethesda I in 7 cases, Bethesda II in 46 cases, Bethesda III in 45 cases, Bethesda IV in 13 cases, Bethesda V in 2 cases and Bethesda VI in 1 case.

**Table 1:** Nodule characteristics. Data are presented as mean ± S.D. or as *n* (%).

Nodule characteristics	All	Nodules not operated	Nodules operated
Number of nodules	1010	669	341
Median size (mm)	24.4 ± 11.8	22 ± 11.9	21 ± 11.7
Laterality, *n* (%)			
Right	508 (50.3)	348 (52)	160 (46.9)
Left	465 (46)	299 (44.7)	166 (48.7)
Isthmus	37 (3.7)	22 (3.3)	15 (4.4)
EU-TIRADS score, *n* (%)			
2	30 (3.0)	25 (3.7)	5 (1.5)
3	338 (33.5)	253 (37.8)	85 (24.9)
4	423 (41.9)	277 (41.4)	146 (42.8)
5	219 (21.7)	114 (17.0)	105 (30.8)
Bethesda score, *n* (%)			
I	84 (8.3)	65 (9.7)	19 (5.6)
II	489 (48.4)	438 (65.5)	51 (15.0)
III	174 (17.2)	112 (16.7)	62 (18.2)
IV	168 (16.6)	44 (6.6)	124 (36.4)
V	30 (3.0)	1 (0.1)	29 (8.5)
VI	65 (6.4)	9 (1.3)	56 (16.4)

### Surgery

Surgery was performed in 287 (33.3%) patients with 341 (33.8%) nodules of which 36% were Bethesda III nodules, 74% were Bethesda IV nodules and 97% were Bethesda V nodules. Surgery consisted of lobectomy in 163 (56.8%) cases and total thyroidectomy in 124 (43.2%) cases. Based on the final pathology of the nodule, a completion thyroidectomy was performed in 12 cases. Final pathology was benign in 219 (64.2%) cases, low-risk neoplasm in 21 (6.2%) cases (NIFTP: 19 cases, TUMP: 1 case, hyalinizing trabecular tumor: 1 case) and malignant in 101 (29.6%) cases (papillary thyroid cancer: 79 cases; follicular thyroid cancer: 10 cases; oncocytic cancer: 5 cases; poorly differentiated thyroid cancer: 5 cases; medullary thyroid cancer: 1 case and intrathyroidal metastasis from renal carcinoma not diagnosed at cytology :1 case).

Rates of malignancy (malignancy only and malignancy plus low-risk neoplasm) in the operated and all nodules according to EU-TIRADS classification and Bethesda classification are detailed in Tables 2, 3, and 4 and Supplementary Table 1 (see section on supplementary materials given at the end of this article). Rate of malignancy in operated nodules, and including cancer only was 0% in EU-TIRADS 2 nodules, 11.9% in EU-TIRADS 3, 32.3% in EU-TIRADS 4, and 55.2% in EU-TIRADS 5 nodules. These rates were 0% for Bethesda I nodules, 2% for Bethesda II, 16.1% for Bethesda III, 14.5% for Bethesda IV, 58.6% for Bethesda V, and 98.2% for Bethesda VI nodules. Rates of malignancy, considering cancer only, among operated nodules that had 1, 2, 3, or 4 signs of EU-TIRADS 5 score were 48%, 44%, 75%, and 100%.

Combining EU-TIRADS score with Bethesda classification only slightly changed the rate of malignancy for Bethesda III, IV, and V nodules with a rate of malignancy in case of EUTIRADS 5 nodules compared to EUTIRADS 3 nodules of 7% vs 4% for Bethesda III cytology for all nodules undergoing FNAC and 19% vs 11% for all nodules undergoing surgery, 21% vs 14% for Bethesda IV cytology for all nodules undergoing FNAC and 29% vs. 21% for all nodules undergoing surgery, 75% vs 71% for Bethesda V cytology for all nodules undergoing FNAC and 75% vs 83% for all nodules undergoing surgery. Those changes were, however, nonsignificant when evaluated with Fisher’s exact test (*P*-value: 0.7 for Bethesda III and IV classes, 1 for Bethesda V class.

**Figure 2:**
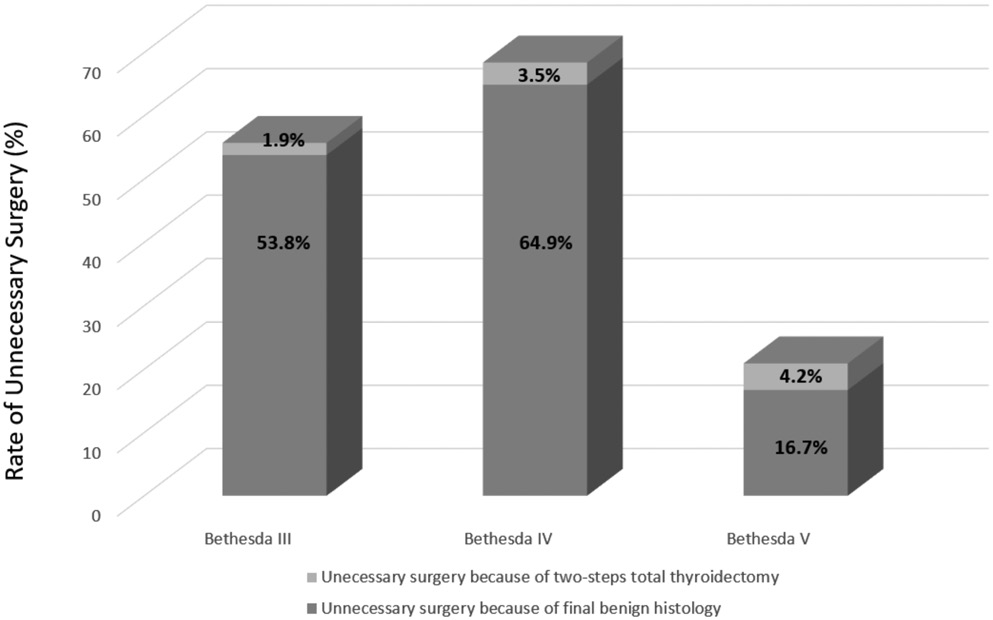
Rates of unnecessary surgery.

Unnecessary surgery for Bethesda III, IV, and V nodules with final benign histology occurred in 28 (53.8%), 74 (64.9%), and four (16.7%) patients, respectively. Two-stage completion thyroidectomy after lobectomy for Bethesda III, IV, and V nodules was required in one (1.9%), four (3.5%), and one (4.2%) patients, respectively (Fig. 2).

**Table 2:** Rates of malignancy (ROM) according to EU-TIRADS and Bethesda scores.

Nodule characteristics		Final pathology of nodules, *n*			ROM in operated nodules, %		ROM in FNAC nodules, %	
	*n* operated/total *n* (%)	Benign	LRN	Malignant	Malignant + LRN	Malignant only	Malignant + LRN	Malignant only
EU-TIRADS score								
2	5/30 (16.7)	5	0	0	0	0	0	0
3	85/338 (25.1)	70	5	10	17.6	11.9	4.4	3.0
4	146/423 (34.5)	102	11	33	30.1	32.3	10.4	7.8
5	105/219 (47.9)	42	5	58	60	55.2	28.8	26.5
Bethesda score								
I	19/84 (22.6)	19	0	0	0	0	0	0
II	51/489 (10.4)	50	0	1	2.0	2.0	0.2	0.2
III	62/174 (35.6)	50	2	10	19.3	16.1	6.9	5.7
IV	124/168 (73.8)	94	12	18	24.2	14.5	17.9	10.7
V	29/30 (96.7)	6	6	17	79.3	58.6	76.7	65.7
VI	56/65 (86.2)**	0	1*	55	100	98.2	86.2	84.6

*Trabecular hyalinizing tumor; **Reasons for the absence of surgery: anaplastic thyroid cancer on cytology (one case), active surveillance (one case), patient refusal (one case), and polymorbid condition or aggressive active extrathyroid malignancy (six patients).

LRN, low-risk neoplasm.

**Table 3:** Rates of malignancy (ROM) according to signs of EU-TIRADS 5 score.

Nodule characteristics		Final pathology of nodules, *n*			ROM in operated nodules		ROM in FNAB nodules	
	*n* operated/total *n* (%)	Benign	LRN*	Malignant	Malignant + LRN	Malignant only	Malignant + LRN	Malignant only
EU-TIRADS 5								
Taller than wide	16/37 (43.2%)	3	0	13	13/16 (81.3%)	13/16 (81.3%)	13/37 (35.1%)	13/37 (35.1%)
Irregular border	78/150 (52%)	30	2	46	48/78 (61.5%)	46/78 (59%)	48/150 (32%)	46/150 (30.7%)
Microcalcifications	36/57 (63.2%)	9	1	26	27/36 (75%)	26/36 (72.2%)	27/57 (47.4%)	26/57 (45.6%)
Hypoechoic	88/163 (54%)	35	5	48	53/88 (60.2%)	48/88 (54.5%)	53/163 (32.5%)	48/163 (29.4%)
EU-TIRADS 5								
1 sign	29/83 (35%)	13	2	14	16/29 (55.2%)	14/29 (48.3%)	16/83 (19.3%)	14/83 (16.9%)
2 signs	45/90 (50%)	22	3	20	23/45 (51.1%)	20/45 (44.4%)	23/90 (25.6%)	20/90 (22.2%)
3 signs	28/43 (65.1%)	7	0	21	21/28 (75%)	21/28 (75%)	21/43 (48.8%)	21/43 (48.8%)
4 signs	4/4 (100%)	0	0	4	4/4 (100%)	4/4 (100 %)	4/4 (100 %)	4/4 (100%)

*LRN: noninvasive follicular thyroid neoplasms with papillary-like features (NIFTP), thyroid tumors of uncertain malignant potential (TUMP), and trabecular hyalinizing tumors.

LRN, Low-risk neoplasm nodules.

**Table 4:** ROM (cancer + low risk neoplasm) according to EU-TIRADS score and Bethesda classification in operated nodules and FNAC nodules.

**Bethesda score**		**EU-TIRADS score**				
	***n***	**All**	**2**	**3**	**4**	**5**
Operated nodules						
I	19	0/19 (0%)	0/2 (0%)	0/5 (0%)	0/8 (0%)	0/4 (0%)
II	51	1/51 (2%)	0/2 (0%)	0/20 (0%)	1/26 (3.8%)	0/3 (0%)
III	62	12/62 (19.4%)	0/1 (0%)	2/19 (10.5%)	7/26 (26.9%)	3/16 (18.8%)
IV	124	30/124 (24.2%)	–	7/34 (20.6%)	15/62 (24.2%)	8/28 (28.6%)
V	29	23/29 (79.3%)	–	5/6 (83.3%)	12/15 (80%)	6/8 (75%)
VI	56	56/56 (100%)	–	1/1 (100%)	9/9 (100%)	46/46 (100%)
FNAC nodules						
I	84	0/84 (0%)	0/15 (0%)	0/23 (0%)	0/28 (0%)	0/18 (0%)
II	489	1/489 (0.2%)	0/14 (0%)	0/208 (0%)	1/209 (0.5%)	0/58 (0%)
III	174	12/174 (6.9%)	0/1 (0%)	2/50 (4%)	7/79 (8.9%)	3/44 (6.8%)
IV	168	30/168 (17.9%)	–	7/49 (14.3%)	15/81 (18.5%)	8/38 (21.1%)
V	30	23/30 (76.7%)	–	5/7 (71.4%)	12/15 (80%)	6/8 (75%)
VI	65	56/65 (86.2%)	–	1/1 (100%)	9/11 (81.8%)	46/53 (86.8%)

## Discussion

The malignancy rate of thyroid nodules varies from 1 to 10%, depending mainly on patient recruitment and the gold standard used to assess malignancy (20, 21, 22). In the absence of surgery, when the exclusion of malignancy is based on the absence of change in the thyroid nodule size on a 6-month follow-up neck US, the rate of malignancy is underestimated. When malignancy is considered in all Bethesda III, IV, V, or VI findings the rate of malignancy is, of course, overestimated. Finally, when malignancy rates are based on postoperative pathology, they are overestimated since nodules selected for surgery are more likely to be malignant and have a higher US rate for malignancy than nodules that do not undergo surgery. Knowing these difficulties, we chose, in our study, to calculate the rate of malignancy in all nodules undergoing FNAC and also only among nodules that underwent surgery. We also chose to calculate rates of malignancy taking into account cancer only, as well as cancer and low-risk neoplasms (including NIFTP and TUMP), since recommendations are to operate these tumors (19).

The malignancy rates in this study are comparable to those reported in the literature (17). In EU-TIRADS 2 nodules, the rate of malignancy (including cancer and low-risk neoplasms) was 0%. In EU-TIRADS 3 nodules, the rate of malignancy was 18% among nodules undergoing surgery and 4% among all nodules undergoing FNAC. These rates were 30% and 10% respectively, for EU-TIRADS 4 nodules, and 60% and 29%, respectively, for EU-TIRADS 5 nodules. In Bethesda I nodules, the rate of malignancy (including cancer and low-risk neoplasms) was 0%. In Bethesda II nodules, this rate was 2% among nodules undergoing surgery and 0.2% among all nodules undergoing FNAC. These rates were 19% and 7% respectively in Bethesda III nodules, 24% and 18%, respectively in Bethesda IV nodules, 79% and 77% respectively in Bethesda V nodules and finally 100% and 86%, respectively, in Bethesda VI nodules. As the rate of malignancy increased from Bethesda III to Bethesda V cytology, the percentage of nodules undergoing surgery increased, and the rates of malignancy among nodules undergoing surgery and among all nodules undergoing FNAC were closer. The situation was different for Bethesda VI nodules because some of the nodules were proposed for active surveillance.

The percentages of Bethesda I and Bethesda III nodules in our cohort were 8.3% and 17.2% respectively, similar to results from literature (5–11% and 2–18% respectively) (4). Rapid on-side evaluation of cytology specimens (ROSE) is found to improve adequacy of samples, and it is systematically performed in our practice (23, 24).

The impact of EU-TIRADS score on malignancy rates in each Bethesda class is a controversial subject (8). There is evidence that specific radiologic features, such as microcalcifications and irregular margins, can improve the diagnostic ability of cytology, but that seems not to be the case for EU-TIRADS classification per se (25). In the present study, combining EU-TIRADS score with Bethesda classification only slightly changed the rate of malignancy for Bethesda III, IV, and V nodules. Those changes were, however, nonsignificant which could be explained by the small numbers of patients in each category.

Bethesda V or VI FNAC in EU-TIRADS 3 nodules is a rare situation, occurring in only 0.8% of nodules. Bethesda II FNAC in EU-TIRADS 5 nodules was more common but still not frequent occurring in only 5.7% of the cohort. This is the basis for the recommendation of a second FNAC in Bethesda II – EU-TIRADS 5 nodules (6, 8). However, the limitation of the EU-TIRADS 5 category is its wide range of malignancy rate. Nodules are classified as EU-TIRADS 5 if at least one of the following signs is present: irregular margins, microcalcification, marked hypoechogenicity, or higher than wide shape. The number of EU-TIRADS 5 signs is, however, highly informative, even though interobserver reproducibility is questionable, with a rate of malignancy of 48% among EU-TIRADS 5 nodules undergoing surgery if only one sign is present, versus 75%, if three signs are present and a rate of 100% if four signs are present (26, 27).

The evaluation of molecular testing costs based on mathematical models and hypotheses must consider the actual management of suspicious FNAC and not only the recommended management, especially in diseases with excellent prognosis, which is the case of most thyroid cancers. In the present cohort, surgery was performed in 36% of Bethesda III nodules, 74% of Bethesda IV nodules and 97% of Bethesda V nodules in the absence of molecular testing, consistent with an increasing risk with increasing Bethesda classification. This also shows that surveillance of Bethesda III and IV nodules is already suggested to patients and is not limited to nodules of 10 mm or less. However, whether this management is relevant remains to be proven. Unnecessary surgery for Bethesda III, IV, and V nodules with final benign histology occurred in 106 (28.5%), a relatively small number. It was more frequent in Bethesda IV category, since more patients underwent surgery in this category. However, 42% of Bethesda III, IV, and V nodules did not undergo surgery when surgery is usually recommended. This brings up the question of the interest in doing FNAC if surgery is not performed even in case of suspicious results. However, a Bethesda II result is reassuring, and knowing the risk of malignancy is the basis for a concrete discussion with patients to decide on management. Of note, two-stage completion thyroidectomy because of after lobectomy was not frequent, being necessary in only 1.9%, 3.5%, and 4.2% of Bethesda III, IV and V nodules, respectively.

Limitations of the present study include its retrospective design, and the absence of follow-up in nonoperated Bethesda III, IV, and V patients. Regarding this latest point, though, assessing the absence of malignancy based on follow-up is never guaranteed to be correct given the slow rate of progression of most thyroid cancers.

In conclusion, in this real data cohort, surgery was unnecessary in more than half of the patients with Bethesda III and IV nodules operated and in 21% of the patients with Bethesda V nodules operated.

## Supplementary Materials

Supplementary Table 1: ROM (Cancer only) according to EU-TIRADS score and Bethesda classification among nodules operated (2A) and among all nodules having FNAC (2B)

## Declaration of interest

F Triponez has received consulting fees from Medtronic and Fluoptics, not related to the present study. S Leboulleux has received consulting fees from Lilly, Bayer, EISAI, not related to the present study. All other authors declare that there is no conflict of interest that could be perceived as prejudicing the impartiality of the research reported.

## Funding

This research did not receive any specific grant from any funding agency in the public, commercial, or not-for-profit sector.

## Author contribution statement

MM and SL conceived the study, collected the data, analyzed the data, and wrote the paper. ES, CDV, AS, MD, PK, and FT analyzed the data and revised the manuscript. EF and FJ revised the manuscript.
