# Supplementary material for: Unnecessary thyroid surgery rate for suspicious nodule in the absence of molecular testing
Source: Eur Thyroid J. 2023 Oct 11;12(6):e230114. doi: 10.1530/ETJ-23-0114 (PMC10620454; doi:10.1530/ETJ-23-0114)
Supplement: Supplementary Table 1: ROM (Cancer only) according to EU-TIRADS score and Bethesda classification among nodules operated (2A) and among all nodules having FNAC (2B) [file supplementary_table_1.pdf]

Supplementary Table 1: ROM (Cancer only) according to EU-TIRADS score and Bethesda classification among nodules operated (2A) and among all nodules having FNAC (2B)

2A

		US EU-TIRADS score			
%	All EU-TIRADS	2	3	4	5
**Bethesda score**					
**I (n=19)**	0% (0/19)	0% (0/2)	0% (0/5)	0% (0/8)	0% (0/4)
**II (n=51)**	2% (1/51)	0% (0/2)	0% (0/20)	3.8% (1/26)	0% (0/3)
**III (n=62)**	16.1% (10/62)	0% (0/1)	10.5% (2/19)	19.2% (5/26)	18.8% (3/16)
**IV (n=124)**	14.5% (18/124)	-	11.8% (4/34)	14.5% (9/62)	17.9% (5/28)
**V (n=29)**	58.6% (17/29)	-	50% (3/6)	60% (9/15)	62.3% (5/8)
**VI (n=56)**	98.2% (55/56)	-	100% (1/1)	100% (9/9)	97.8% (45/46)

2B

		US EU-TIRADS score			
%	All EU-TIRADS	2	3	4	5
**Bethesda score**					
**I (n=84)**	0% (0/84)	0% (0/15)	0% (0/23)	0% (0/28)	0% (0/18)
**II (n=489)**	0.2% (1/489)	0% (0/14)	0% (0/208)	0.5% (1/209)	0% (0/58)
**III (n=174)**	5.7% (10/174)	0% (0/1)	4% (2/50)	6.3% (5/79)	6.8% (3/44)
**IV (n=168)**	10.7% (18/168)	-	8.2% (4/49)	11.1% (9/81)	13.2% (5/38)
**V (n=30)**	56.7% (17/30)	-	42.9% (3/7)	60% (9/15)	62.5% (5/8)
**VI (n=65)**	84.6% (55/65)	-	100% (1/1)	81.8% (9/11)	84.9% (45/53)
